# Pharmacophore-based design and discovery of (−)-meptazinol carbamates as dual modulators of cholinesterase and amyloidogenesis

**DOI:** 10.1080/14756366.2016.1265521

**Published:** 2017-03-08

**Authors:** Qiong Xie, Zhaoxi Zheng, Biyun Shao, Wei Fu, Zheng Xia, Wei Li, Jian Sun, Wei Zheng, Weiwei Zhang, Wei Sheng, Qihong Zhang, Hongzhuan Chen, Hao Wang, Zhuibai Qiu

**Affiliations:** a Department of Medicinal Chemistry, School of Pharmacy, Fudan University, Shanghai, P. R. China;; b Department of Pharmacology, Institute of Medical Sciences, Shanghai Jiao Tong University School of Medicine, Shanghai, P. R. China;; c NPFPC Key Laboratory of Contraceptives and Devices, Shanghai Institute of Planned Parenthood Research, Shanghai, P. R. China

**Keywords:** Pharmacophore, carbamates, (−)-meptazinol, acetylcholinesterase, amyloidogenesis

## Abstract

Multifunctional carbamate-type acetylcholinesterase (AChE) inhibitors with anti-amyloidogenic properties like phenserine are potential therapeutic agents for Alzheimer’s disease (AD). We reported here the design of new carbamates using pharmacophore model strategy to modulate both cholinesterase and amyloidogenesis. A five-feature pharmacophore model was generated based on 25 carbamate-type training set compounds. (−)-Meptazinol carbamates that superimposed well upon the model were designed and synthesized, which exhibited nanomolar AChE inhibitory potency and good anti-amyloidogenic properties in *in vitro* test. The phenylcarbamate **43** was highly potent (IC_50_ 31.6 nM) and slightly selective for AChE, and showed low acute toxicity. In enzyme kinetics assay, **43** exhibited uncompetitive inhibition and reacted by pseudo-irreversible mechanism. **43** also showed amyloid-β (Aβ) lowering effects (51.9% decrease of Aβ_42_) superior to phenserine (31% decrease of total Aβ) in SH-SY5Y-APP_695_ cells at 50 *µ*M. The dual actions of **43** on cholinergic and amyloidogenic pathways indicated potential uses as symptomatic and disease-modifying agents.

## Introduction

Alzheimer’s disease (AD) is an age-related neurodegenerative disorder that causes the majority of dementia in the elderly. Pathologically, AD is characterized by the progressive loss of basal forebrain cholinergic neurons^1^, and neuropathological changes of abnormally accumulated extracellular amyloid-β peptide (Aβ)^2^ and intracellular tau protein^3^. However, the underlying mechanisms of AD are still poorly understood, which may be attributed to the complex multipathogenic features^4^^,^^5^, including amyloidogenic processing of amyloid precursor protein (APP), Aβ aggregation, tau hyperphosphorylation, calcium dyshomeostasis, oxidative stress, mitochondrial dysfunction, deterioration of synaptic neurotransmission, and neuronal apoptosis.

**Figure 1.**
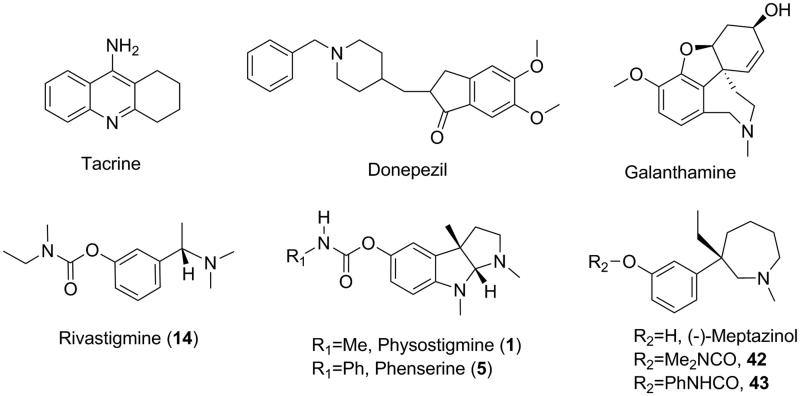
Structures of AChE inhibitor anti-AD drugs, classical carbamate-type AChE inhibitors and (−)-meptazinol carbamates.

Current approved anti-AD drugs are all palliative treatments targeting cholinergic or glutamatergic neurotransmission thereby symptomatically improving memory and cognition in patients. Acetylcholinesterase (AChE) inhibitors (Figure 1) such as tacrine, donepezil, rivastigmine, and galanthamine, are major palliative treatments available now. Carbamates are classical pseudo-irreversible AChE inhibitors, which bind to AChE catalytic site covalently via carbamylating conserved serine residue, and therefore delay the reactivation of an unbound enzyme. Physostigmine (**1**) is the first AChE inhibitor separated from natural products, but unacceptable toxicity limits its clinical use. Rivastigmine (**14**) is the only carbamate AChE inhibitor approved as anti-AD drug on the market.

Over the last decade, much effort has been devoted to amyloidogenesis (APP generation/metabolism) and Aβ-induced neurotoxicity. Unfortunately, to date, Aβ-directed therapies, such as γ-secretase inhibitors and immunotherapies^6^, were too toxic to succeed in clinical trials^7^. It seems that the one-molecule-one-target paradigm is inadequate to address the unmet disease-modifying goal of anti-AD drugs. In view of the multifactorial nature of AD pathogenesis, a “multi-target-directed ligands” (MTDLs)^8^ strategy was applied in recent development of modifying treatments for AD^8–10^. Single molecule directing toward different biological targets involved in AD etiology showed promising multipotent profiles. Multifunctional AChE inhibitors with anti-amyloidogenic properties have been most widely studied^11^ because of their symptom-alleviating ability and disease-modifying potential.

Phenserine (**5**)^12^, a phenylcarbamate of (−)-eseroline (**24**), developed by Greig et al.^13^, is a unique multipotent AChE inhibitor. It reduces the levels of APP and Aβ via a non-cholinergic mechanism by down-regulating the translation of APP mRNA^14^. The phenylcarbamoyl moiety of phenserine seems to be crucial for its anti-amyloidogenic effect. Although phase III clinical trials of phenserine failed due to lack of efficacy^15^, a redevelopment after correcting some methodological deficiencies^16^ might bring to new conclusions.

In our earlier research, bis-(−)-*nor*-meptazinols^17–19^ and their derivatives^20^ were characterized as dual binding site AChE inhibitors with anti-Aβ-aggregation and/or metal-complexing properties. The crystal structure of bis-(−)-*nor*-meptazinol/AChE complex^18^ was resolved, which revealed the binding pose of (−)-meptazinol moiety in the AChE catalytic site. To further explore (−)-meptazinol monomer derivatives, we reported here the design of new carbamates using pharmacophore model strategy with the aim to modulate both AChE activity and amyloidogenesis.

Based on three-dimensional (3D) quantitative structure-activity relationship (QSAR) analysis of 25 known carbamate-type AChE inhibitors^21–29^, a pharmacophore model was built *in silico* and validated through a test set of 16 structurally diverse compounds^22–24^^,^^26^^,^^28^^,^^30–35^. Guided by the pharmacophore model, (−)-meptazinol was selected as the scaffold to build carbamoyl groups on. Herein, (−)-meptazinol carbamates (**42** and **43**, Figure 1) were designed and synthesized, and their AChE inhibitory activities were predicted according to ligand pharmacophore mapping. To illustrate potential complementary interactions of the model features with enzyme residues, the pharmacophore model was fitted into the active site of AChE.

Inhibitory potencies of **42** and **43** for AChE and butyrylcholinesterase (BChE) were tested *in vitro*. Enzyme kinetic parameters, Michaelis constant (*K*_m_) and maximum velocity of reaction (*V*_max_), were measured on recombinant human acetylcholinesterase (rHuAChE). The association and dissociation rate constants, namely inhibit constant (*k*_i_), dissociation constant (*k*_3_), and affinity constant (*K*_D_), were determined using AChE immobilized disk. Anti-amyloidogenic experiments were conducted employing high content screening (HCS) in SH-SY5Y-APP_695_ cells, and enzyme-linked immunosorbent assay (ELISA) in the cell culture medium. Mechanisms for the actions of **42** and **43** on reducing APP and Aβ_42_ levels were further discussed.

## Materials and methods

### Pharmacophore modeling

Pharmacophore model generation and validation were performed using 3D QSAR Pharmacophore Generation module and Ligand Pharmacophore Mapping module, respectively, in Discovery Studio v2.5 (DS, Accelrys, San Diego, CA) software package. Carbamate-type AChE inhibitors with comparable IC_50_s tested by Ellman’s method^36^ and using physostigmine or rivastigmine as positive control were collected from the literature^21–29^ to generate quantitative pharmacophore hypotheses. The IC_50_ values covered a range of three to four orders of magnitude and the activity uncertainty was set 3 as default. All two-dimensional (2D) structures of the compounds were built using ISIS Draw v2.2 (MDL Information Systems, Inc.) and exported into DS to be converted into 3D format. A maximum of 255 conformers were generated for each compound over a 20 kcal/mol range using the BEST conformational analysis method. As an exception, (−)-meptazinol and its carbamate derivatives were calculated by both BEST and CAESAR conformation algorithms. Chemical features including hydrogen bond acceptor (HBA), ring aromatic (RA), positive ionizable (PI) and hydrophobic (HYD) features were selected and each feature was given parameters from a minimum of 1 to a maximum of 5. The minimum interfeature distance was set as a value of 2.50 and the maximum excluded volumes was set to 5. Different weights were assigned to the features and weight variation was the default value of 0.302. Otherwise default parameters were used.

### Chemistry

#### General

All reagents except phenyl isocyanate were of commercial quality. Phenyl isocyanate was prepared from aniline and bis(trichloromethyl) carbonate. Rivastigmine hydrochloride standard was available from Sunve (Shanghai) Pharmaceutical Co., Ltd. Melting points were measured in open glass capillary tubes with Thiele-Dennis tube, and were uncorrected. Specific rotation ([*α*]_D_) was determined on a JASCOP-1020 rotatory apparatus. IR data were recorded on an AVATAR 360 FT-IR spectrometer (KBr). NMR data were recorded on a Mercury Plus 400 instrument. Chemical shifts (*δ*) are expressed in parts per million (ppm), relative to tetramethylsilane (TMS) as an internal standard. Signals of active hydrogen disappeared after D_2_O exchange. Mass spectrum was measured on an Agilent 1100 Series LC/MSD 1946D spectrometer. Elemental analysis was tested on vario EL III element analyzer. Purity of the target compound was verified via HPLC. The elution with acetonitrile-0.01 mol/L KH_2_PO_4_ (pH =4.0) (33:67) was running through a Diamonsil^®^ C18(2) (200 × 4.6 mm, 5 µm) column at a flow rate of 1.0 mL/min and at the temperature of 30 °C using UV detection at 233 nm.

#### Synthesis of (S)-3-(3-ethyl-1-methylazepan-3-yl)phenyl dimethylcarbamate (42)

To a cooled and stirred mixture of 80% sodium hydride (0.15 g, 5.00 mmol) in 10 mL dry tetrahydrofuran, a solution of (−)-meptazinol (0.40 g, 1.71 mmol) in 10 mL dry tetrahydrofuran was added dropwise. The mixture was stirred in ice-water bath for 30 min, then N,N-dimethylcarbamoyl chloride (195 µL, 2.06 mmol) was added. After stirring at room temperature for 2 h, solvents were removed under reduced pressure. Then, water (20 mL) was added, and the mixture was extracted with EtOAc (15 mL × 2). Combined organic layer was washed by brine, dried over anhydrous Na_2_SO_4_, and concentrated *in vacuo* to give a whitish oil **42** (0.52 g, 100% yield). Adding dry HCl-ether to a solution of **42** in dry ether (pH adjusted to 4) afforded the hydrochloride salt **42**·HCl (0.56 g, 97% yield). Crystallization of **42**·HCl (0.50 g) from acetone gave needlelike crystals (0.17 g, 34% yield). mp 163–165 °C; [*α*]_D_^20^ = −17.95 (*c* = 0.10, MeOH); ^1^H NMR (400 MHz, DMSO-d_6_, TMS): *δ* 10.17 (br s, 1/2 H, NH^+^), 8.62 (br s, 1/2 H, NH^+^), 7.43–7.35 (m, H, Ar-H), 7.26–6.99 (m, 3H, Ar-H), 3.98–3.58 (m, H, N-CH_2_), 3.48–3.38 (m, H, N-CH_2_), 3.18–3.04 (m, 5H, N-CH_2_, N-CH_3_), 2.90 (d, 3H, CON-CH_3_, *J* = 3.72 Hz), 2.83 (d, 3H, CON-CH_3_, *J* = 4.69 Hz), 2.41–1.41 (m, 8H, CH_2_), 0.52–0.47 ppm (m, 3H, CH_3_); 13C NMR (DMSO-d_6_): 154.0 (C = O), 151.7 & 151.5 (C_Ar_), 145.3 & 143.8 (C_Ar_), 129.3 & 129.1 (C_Ar_), 123.6 & 123.1 (C_Ar_), 120.5 (C_Ar_), 120.2 (C_Ar_), 66.2 & 63.0 (NCH_2_), 59.5 & 57.9 (NCH_2_), 47.1 & 46.4 (NCH_3_), 44.1 & 43.8 (C), 36.3 (CON-CH_3_), 36.1 (CON-CH_3_), 35.9 & 35.3 (CH_2_), 33.5 & 32.9 (CH_2_), 26.3 & 24.7 (CH_2_), 20.6 & 20.5 (CH_2_), 8.2 & 8.0 (CH_3_); MS (ESI): *m/z* 305.2 [M + H]^+^; Anal. C_18_H_28_N_2_O_2_•HCl•1/4H_2_O (C, H, N).

#### Synthesis of (S)-3-(3-ethyl-1-methylazepan-3-yl)phenyl phenylcarbamate (43)

(−)-Meptazinol (0.40 g, 1.71 mmol) was dissolved in anhydrous ether (15 mL), and a piece of Na metal (approximately 5 mg) was added. The mixture was stirred under nitrogen at room temperature for 10 min, then phenyl isocyanate (233 µL, 2.13 mmol) was added. The reaction mixture was stirred at room temperature for 3 h till the starting material had disappeared. 5 mL of H_2_O were added to destroy any trace of remaining unreacted phenylisocyanate and pH was adjusted to 3 by adding 1N HCl. The mixture was washed with ether (10 mL × 3), basified with saturated Na_2_CO_3_ aqueous solution (adjusting pH to 9), and then extracted with ether (10 mL × 3). The latter ether layer was washed with brine, dried over anhydrous Na_2_SO_4_, and filtered to obtain a clear ether solution of the product. Evaporation of the solvent gave **43** (0.43 g, 72% yield) as a white solid. Acidification of **43** in dry ether using HCl-ether (adjusting pH to 4) afforded **43** hydrochloride as a white powder (0.39 g, 82% yield): mp 122–127 °C; [*α*]_D_^20^ = −18.94 (*c* = 0.108, MeOH); ^1^H NMR (400 MHz, DMSO-d_6_, TMS): *δ* 10.23 (s, H, CONH), 9.98 (br s, 1/2 H, NH^+^), 8.59 (br s, 1/2 H, NH^+^), 7.50–7.40 (m, 3H, Ar-H), 7.33–7.21 (m, 3.5H, ArH), 7.16–7.10 (m, 1.5H, ArH), 7.04 (t, *J* = 7.43 Hz, H, Ar-H), 4.00–3.60 (m, H, N-CH_2_), 3.50–3.40 (m, H, N–CH_2_), 3.11 (m, 2H, N–CH_2_), 2.84 (m, 3H, N–CH_3_), 2.19–1.43 (m, 8H, CH_2_), 0.51 ppm (t, *J* = 7.04 Hz, 3H, CH_3_); 13C NMR (DMSO-d_6_): 151.7 (C = O), 150.9 & 150.7 (C_Ar_), 145.5 & 144.0 (C_Ar_), 138.6 (C_Ar_), 129.6 & 129.4 (C_Ar_), 128.9 (2 C_Ar_), 123.9 & 123.4 (C_Ar_), 123.0 (C_Ar_), 120.6 (C_Ar_), 120.3 (C_Ar_), 120.2 (C_Ar_), 118.5 (C_Ar_), 66.2 & 62.9 (NCH_2_), 59.5 & 58.0 (NCH_2_), 47.1 & 46.4 (NCH_3_), 44.2 & 43.9 (C), 35.9 & 35.4 (CH_2_), 33.5 & 33.0 (CH_2_), 26.3 & 24.8 (CH_2_), 20.6 & 20.4 (CH_2_), 8.2 & 8.0 (CH_3_); MS (ESI): *m/z* 353.2 [M + H]^+^; Anal. C_22_H_28_N_2_O_2_•HCl•1/2H_2_O (C, H, N). HPLC: *t*_R_ = 8.5 min, 98.4% purity.

### *In vitro* AChE/BChE inhibition assays

Inhibitory activities of the compounds toward AChE and BChE were evaluated by Ellman’s method^36^, employing mice brain homogenate as source of AChE and mice serum as source of BChE. Briefly, 270 µL of a solution of AChE (1:9 w/v homogenate in 0.1 M phosphate buffer (PB), pH 7.4) and 30 µL of a solution of the tested compound (**42**, **43**, or rivastigmine, six to seven concentrations) were mixed adequately. After incubation for 20 min at 37 °C, Ellman’s reagent (300 *µ*L, 5,5′-dithiobis(2-nitrobenzoic acid) (DTNB), 0.5 mM in 0.1 M PB, pH 7.4) and acetylthiocholine iodide (ATCh) (300 *µ*L of 0.5 mM water solution) were added successively, and percent inhibition was determined by absorbance changes at 412 nm detected by UV spectrophotometry compared with control. BChE inhibition assay was similarly carried out using butyrylthiocholine iodide (BTCh) (0.5 mM) as the substrate and BChE (1:19 v/v serum in 0.1 M PB, pH 7.4) as the enzyme source. The concentration of a compound that produced 50% inhibition of the enzyme activity, namely IC_50_ value, was calculated by nonlinear least squares regression of the response-concentration (log) curve. Results are reported as the mean ± SEM (standard error of the mean) of IC_50_ obtained from at least three independent measures^38^.

### Determination of the enzyme kinetic parameters K_m_ and V_max_

10 µL inhibitors of different concentrations and 50 µL rHuAChE enzyme solution of 0.5 U/mL were mixed and incubated for 20 min, then 75 µL of DTNB solution and 100 µL of ATCh solution (concentration ranging from 0.057 mM to 0.2 mM) were added to the mixture. Enzyme activity was determined right after ATCh was added by modified Ellman’s spectrophotometrical method^37^. The *K*_m_ and *V*_max_ values for AChE inhibition were calculated by regression analysis of Lineweaver–Burk plots (1/velocity versus 1/[substrate]).

### Determination of carbamoylation and decarbamoylation rate constants

**Scheme 1.**
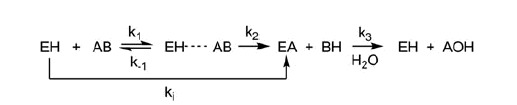
The process of AChE carbamoylation and decarbamoylation.^a^^a^EH is the free enzyme, AB is the inhibitor, EH · · · AB is the non-covalent complex and EA is the carbamylated enzyme. *k*_i_ is inhibit constant and *k*_3_ is dissociation constant.

The EDA CIM disk was first connected to a syringe pump and equilibrated with 10 column volumes (CV) of triple-distilled water and 10 CVs of 0.5 M Na-PB (pH 8.0). The flow rate was set at 0.5 mL/min. Then, the disk was activated by a 1% glutaraldehyde solution in PB (0.1 M, pH 7.4) (in the dark, 12 h, 4 °C). The activated disk was washed with 10 CVs of PB. 2 mL of rHuAChE in PB (10 U/mL) was then added into the column and left to react overnight (4 °C). The Schiff bases were reduced by 5 mL of 0.1 M sodium triacetoxyborohydride (STAB) in PB (2 h at room temperature) and 5 mL of 0.2 M monoethanolamine in PB (3 h at room temperature)^39^. The column was connected to the liquid chromatography system and equilibrated with a mobile phase consisting of 0.5 M NaCl and 10^−4^ M DTNB in PB (buffer A) for 10 min. Reference enzyme activity was assessed by injecting 20 µL of saturating substrate (ATCh, 50 mM). The peak area was calculated and noted as *A*_0_. Then, the mobile phase was switched to the one containing selected concentrations of inhibitor (carbamoylation phase). Aliquots of 20 µL of saturating substrate were injected every 10–20 min and the time-dependent decreasing of peak area (*A*_i_) was monitored down to a plateau. Once a constant plateau was reached, mobile phase was switched to buffer A again (decarbamoylation phase). Aliquots of substrate were kept injecting into the system to monitor the recovery of AChE activity over time. Percent inhibition of enzyme activity [(*A*_0_−*A*_i_)/*A*_0_×100%] was plotted versus time. The experiment was carried out for two different concentrations of **42** and **43** (50 and 100 nM). The process of AChE carbamoylation and decarbamoylation is generally described as Scheme 1^40–42^.

Calculation of *k*_i_ and *k*_3_ was performed by applying Perola’s mathematical equation^43^. The affinity of the inhibitors toward the enzyme (*K*_D_) was calculated following Equation (1).

(1) $$K_{\text{D}}=\frac{k_{\text{i}}}{k_{\text{3}}}$$

### Quantification of APP by high content screening (HCS cellomics)

Human neuroblastoma SH-SY5Y cells transfected with wild-type APP_695_ gene (SH-SY5Y-APP_695_)^44^ of 5 × 10^4^/well (SH-SY5Y-APP_695_ kindly provided by Prof. Shengdi Chen, Ruijin Hospital, Shanghai Jiao Tong University School of Medicine) were plated in 200 µL culture medium (DMEM/F12, 10% Fetal Bovine Serum, 300 *µ*g/mL G418, 100 U/mL penicillin + 100 *µ*g/mL streptomycin) and incubated for 48 h at 37 °C in 5% CO_2_. Compounds **42** and **43** dissolved in culture medium were applied to the cells and incubated at 37 °C in 5% CO_2_ for 16 h. The cells were fixed and permeabilized. Mouse anti-APP monoclonal antibody N-terminus (MAB348, CHEMICON) and Goat anti-Mouse IgG conjugated to Alexa Fluor 555 were used as primary antibody and secondary antibody to detect APP while Hoechst Dye was used to stain nucleus simultaneously. KineticScan HCS System was employed to automatically find, focus, image, and analyze the cells on double-fluorescence channel guided by Target Activation Bioapplication. Fluorescence Unit of APP versus that of nucleus was used to evaluate the expression of APP, in order to balance the background.

### Quantification of Aβ_40_/Aβ_42_ by ELISA

The cell culture medium was collected and added with phenylmethylsulfonyl fluoride (PMSF) to the final concentration of 0.1%. The concentration of Aβ_40_/Aβ_42_ in the medium was measured by Human/Rat βAmyloid (**40/42**) ELISA Kit Wako and calculated according to the standard line.

### Acute toxicity test

Acute toxicity was evaluated in mice of both sexes (20–25 g Kunming mice from experimental animal center of Shanghai Jiao Tong University School of Medicine). All animals were housed in plastic cages with food and water *ad libitum* and maintained on a 12/12 h light/dark cycle at 22 ± 1 °C. They were randomly assigned to one of the five concentrations between 0% and 100% lethal rate according to our preliminary studies (data not shown). Compounds **42** and **43** were dissolved in a 1:10 propylene glycol-normal saline mixture at 0.1 mol/L and then diluted to the final stepwise concentrations with normal saline. Each concentration of the compound was orally or intraperitoneally administered to a group of 10 animals. After two weeks of observation, the lethal rate for each group was measured. A 95% confidence interval for **42** or **43** after oral or intraperitoneal administration was calculated by Bliss method.

## Results and discussion

### 3D pharmacophore generation

To find out common structural elements necessary for AChE inhibition, quantitative 3D pharmacophore modeling was performed *in silico* using Discovery Studio v2.5 (DS, Accelrys, San Diego, CA) on 25 carbamate-type AChE inhibitors with diverse scaffolds. Carbamates with comparable IC_50_s tested by Ellman’s method^36^ and using physostigmine or rivastigmine as positive control were collected from the literature^21–29^. As illustrated in Table 1, different physostigmine derivatives (A)^22–24^, enantiomers (B)^22^^,^^25^, and 8-carba analogs (C)^26^ had been enrolled into the training set (**1–11**). Rivastigmine derivatives (D)^27^ and conformationally restricted closed-ring rivastigmine analogs with benzopyrano[4,3-b]pyrrole (E)^28^, aminoindane (F)^29^ and aminotetralin (G)^29^ scaffolds had also been included (**12–23**). Phenol compounds without carbamoyl group, such as (−)-eseroline (**24**)^21^ and (**25**)^28^, fell into the category of inactive compounds. IC_50_ values of all the training set compounds covered a range of three to four orders of magnitude, spanning from 8 nM to 40 µM (Table 1).

**Table 1.** Structures of training set molecules (**1–25**), their experimental IC_50_s reported in the literature, and estimated IC_50_s based on fit values to the pharmacophore model.^a^


	Substituents										
Cpd.	Isomer/Pos^b^	R^1^	R^2^	R^3^	R^4^/X	R^5^	Experimental IC_50_ (nM)^c^	Estimated IC_50_ (nM)	Fit value	Error^d^	Reference^e^
**1**	(−)	Me	H	Me	Me	Me	28	23	8.75	−1.2	22
**2**	(+)	Me	H	Me	Me	Me	9900	2000	6.80	−4.8	22
**3**	(−)	Me	H	Me	H	H	11	21	8.79	+1.9	22
**4**	(+)	Me	H	Me	H	H	1500	1300	6.99	−1.1	22
**5**	(−)	Ph	H	Me	Me	Me	24	19	8.83	−1.3	22
**6**	(+)	Ph	H	Me	Me	Me	3500	5100	6.40	+1.5	22
**7**	(−)	2′Me-Ph	H	Me	Me	Me	10	17	8.88	+1.7	23
**8**	(+)	2′Me-Ph	H	Me	Me	Me	5500	1500	6.92	−3.6	25
**9**	(−)	Me	H	Ph	Me	Me	9300	3100	6.62	−3.0	24
**10**	(−)	Me	H	Me	Et		38	31	8.61	−1.2	26
**11**	(−)	Me	H	H	Et		250	320	7.60	+1.3	26
**12**		Me	H				13^f^	63	8.31	+4.8	27
**13**		Me	Me				27^f^	93	8.14	+3.5	27
**14**		Me	Et				3000^f^	1900	6.83	−1.6	27
**15**	6-	Me	H		S		8	8	9.20	1.0	28
**16**	6-	Me	H		CH_2_		17	13	8.99	−1.3	28
**17**	6-	Me	H		O		30	12	9.02	−2.5	28
**18**	7-	Me	H		O		1900	1600	6.91	−1.2	28
**19**	8-	Me	H		O		16 000	5700	6.35	−2.9	28
**20**	5-	Me	Me				760	1300	7.01	+1.7	29
**21**	4-	Me	Me				460	4600	6.44	+10	29
**22**	8-	Me	Me				1500	1600	6.91	+1.1	29
**23**	7-	Me	Me				3200	4900	6.41	+1.5	29
**24**	(−)						>10 000	12 000	6.04	+1.2	21
**25**	(−)						40 000	36 000	5.55	−1.1	28

a Hypo cost = 116.65, Fixed cost = 108.67, Null cost =162.82. Statistic parameters of the model and the training set: cost difference (Δcost = 46.16); root mean square (RMS = 0.78; correlation coefficient (*r* = 0.95).

b Position where carbamoyloxyl groups substituted.

c AChE from human erythrocytes was used unless otherwise indicated.

d Ratio between estimated and experimental IC_50_ values. “+” indicates that the estimated IC_50_ is higher than the experimental IC_50_; “−” indicates that the estimated IC_50_ is lower than the experimental IC_50_; a value of 1 indicates that the estimated IC_50_ is equal to the experimental IC_50_.

e References to the literatures that reported the experimental AChE inhibitory activities.

f In this special case, mice brain AChE was used.

3D QSAR Pharmacophore Generation module was used to build pharmacophore models based on HBA, RA, PI and HYD features. Top 10 resultant pharmacophore hypotheses were generated and statistical parameters were used to select the best pharmacophore model. The best pharmacophore model should have the highest cost difference, lowest root mean square (RMS), and best correlation coefficient. Fixed and null costs are two important theoretical cost values to evaluate pharmacophore hypotheses, and the difference between null and fixed cost (cost difference) represents the goodness of a pharmacophore model. A cost difference of 40–60 means a predictive correlation probability of 75–90%. Correlation coefficient is based on linear regression of experimental versus estimated activities. In this study, top-ranked hypothesis was selected as the best pharmacophore model due to the highest cost difference (Δcost: 46.16), lowest RMS (RMS: 0.78), and best correlation coefficient (*r*: 0.95) (Table 1).

Experimental and estimated activities, fit values, and corresponding error values of the training set molecules are listed in Table 1. Error is the ratio between estimated activity and experimental activity. Estimated activity is predicted based on fit value. Fit value indicates how exactly structural components in a molecule are localized in the center of pharmacophoric feature spheres, and thus represents how well a molecular conformation matches the pharmacophore model. All the training set compounds were predicted in their same order of magnitude except that compound **21** was underestimated with an error of +10. The most active compound **15** (IC_50_ 8 nM) had a fit value of 9.20, whereas the least active compound **25** (IC_50_ 40 000 nM) showed lesser value of 5.55.

**Figure 2.**
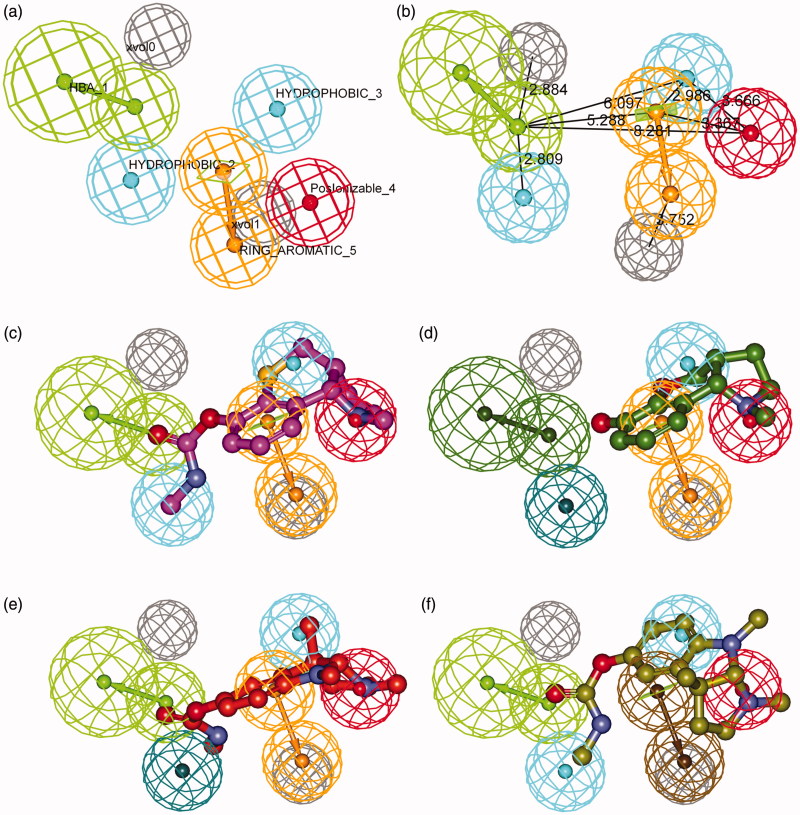
Details of the best pharmacophore model (**a**, **b**) and mapping of representative training set compounds to the pharmacophore model (**c**, **d**, **e**, **f**). (**a**) Spatial disposition of the pharmacophoric features. Hydrogen bond acceptor (HBA, green), ring aromatic (RA, orange), positive ionizable (PI, red), hydrophobic (HYD, cyan) and excluded volume (Xvol, gray). Weights: HBA_1 (3.18); HYD_2 (1.39); HYD_3 (2.58); PI_4 (1.99); RA_5 (1.99). (**b**) Distances between feature centers. (**c**) Mapping of the most active compound (**15**, IC_50_ 8 nM); (**d**) Mapping of the least active compound (**25**, IC_50_ 40000 nM); (**e**) Mapping of an eutomer (3aS)-(−)-physostigmine (**1**, IC_50_ 28 nM); (**f**) Mapping of a distomer (3aR)-(+)-physostigmine (**2**, IC_50_ 9900 nM). Dark-colored (dark green, dark blue, and brown) spheres indicate unmatched features.

The best pharmacophore model consists of five chemical features (HBA, RA, PI, and two HYD features) and two excluded volumes (Figure 2(a,b)). A pair of green spheres indicates one HBA feature with an arrow showing the direction of hydrogen bond. A pair of orange spheres indicates one RA feature with an arrow showing a normal to the aromatic plane. Red sphere stands for PI feature, and cyan ones are indicative of two HYD regions. Gray spheres represent two excluded volumes that unfavorable steric effects may occur. Spatial disposition of the model features was described in Figure 2(a) and distances between feature centers were labeled in Figure 2(b). Figure 2(c–f) illustrated the mapping of representative training set compounds to the pharmacophore model.

Features in the pharmacophore model were assigned different weights, which indicated varied importance. HBA (weight: 3.18) is inevitably the most important feature since carbamoyl group is the basis of covalent carbamylation. Thus phenol compound **25** (Figure 2(d)), which failed to fit the HBA feature, showed very low activity; whereas corresponding methylcarbamate **15** (Figure 2(c)) was the most active compound. PI and RA (weight: 1.99) were mapped to amino and phenyl groups, respectively. Protonated amine interacts with aromatic residues in the catalytic site via cation-π interactions, which stabilizes the transition state of inhibitor-enzyme conjugate in the process of carbamylation. The distance between HBA and PI, namely O-N distance between the oxygen of carbonyl and the nitrogen of amine, plays an important role in the inhibition of AChE for carbamate-type compounds. As Figure 2(b) illustrated, the optimal distance between HBA and PI was 8.281 Å, indicating a range from 7.281 to 9.281 Å.

Unexpectedly, a small but important region of HYD (weight: 2.58) was identified in the model very near to the PI and RA features, which explained the activity difference between enantiomers. Figure 2(e) showed the alignment of physostigmine (**1**) to the pharmacophore model. Carbamoyl group and phenol ring overlapped to HBA and RA features, respectively. Nitrogen atom in 1-position instead of 8-position was aligned to the PI feature. (3aS)-methyl group in (−)-physostigmine (**1**) perfectly matched the HYD region (Figure 2(e)) and thus it shows high potency (IC_50_ 28 nM^22^). In contrast, the R-enantiomer (+)-physostigmine (**2**) is much less potent (IC_50_ 9900 nM^22^). It demonstrated that configurational inversion of the two asymmetric centers (3a and 8a) caused crucial conformational changes which led to pharmacophore model mismatch. When (+)-physostigmine (**2**) yielded to map into the HYD region as shown in Figure 2(f), its phenyl group switched away from the RA feature.

The proposed pharmacophore model was then validated by a test set of 16 compounds^22–24^^,^^26^^,^^28^^,^^30–35^ (**26–41**). Estimated AChE inhibitory activities based on fit values are listed in Table 2. All the test set compounds were predicted in their same order of magnitude. Consequent correlation coefficient of 0.91 for the test set indicated good predictive power of the pharmacophore model.

**Table 2.** Estimated IC_50_s of the test set compounds (**26–41**) compared with their experimental IC_50_s reported in the literature to validate the pharmacophore model.^a^


	Substituents									
Cpd.	R^1^	R^2^	R^3^	R^4^	R^5^	Experimental IC_50_ (nM)^b^	Estimated IC_50_ (nM)	Fit value	Error^c^	Reference^d^
**26**	Me	H	Me	Me	H	57	20	8.80	−2.9	30
**27**	Me	H	Me	Me	H	2200	480	7.43	−4.6	30
**28**	Me	H	Me	H	Me	21	22	8.76	+1.0	22
**29**	Me	H	Me	H	Me	190	760	7.23	+4.0	31
**30**	Ph	H	Me	Me	H	41	18	8.86	−2.3	30
**31**	Ph	H	Me	Me	H	5700	2900	6.64	−2.0	30
**32**	2′Et-Ph	H	Me	Me	Me	10	24	8.73	+2.4	23
**33**	2′iPr-Ph	H	Me	Me	Me	15	26	8.69	+1.7	23
**34**	Ph	H	Ph	Me	Me	10 000	4600	6.45	−2.2	24
**35**	Me	H	Me	Pr	H	150	68	8.28	−2.2	26
**36**	Me	H	H	Pr	H	2000	960	7.12	−2.1	26
**37**						74	160	7.89	+2.2	28
**38**						7	23	8.74	+3.3	32
**39**						680^e^	1600	6.91	+2.4	33
**40**						1200^f^	2900	6.64	+2.4	34
**41**						2200	630	7.31	−3.5	35

a Statistic parameters of the test set: correlation coefficient (*r* = 0.91).

b AChE from human erythrocytes was used unless otherwise indicated.

c Ratio between estimated and experimental IC_50_ values. “+” indicates that the estimated IC_50_ is higher than the experimental IC_50_; “−” indicates that the estimated IC_50_ is lower than the experimental IC_50_; a value of 1 indicates that the estimated IC_50_ is equal to the experimental IC_50_.

d References to the literatures that reported the experimental AChE inhibitory activities.

e Mice brain AChE was used.

f AChE from bovine erythrocytes was used.

### Mapping of the pharmacophore model into AChE active site

**Figure 3.**
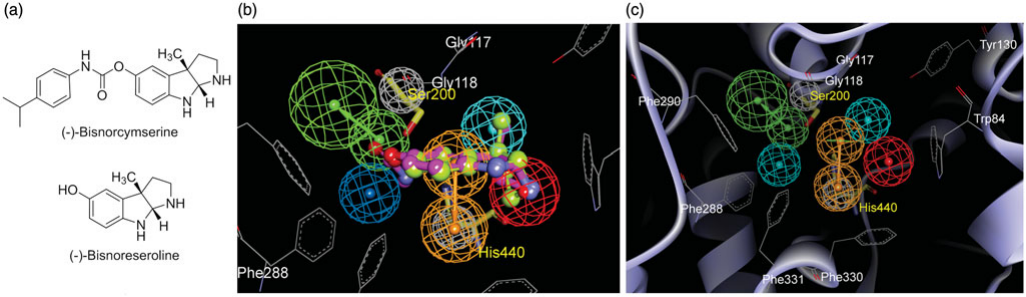
**(a**) Structures of (−)-bisnorcymserine and (−)-bisnoreseroline; (**b**) Superimposition of the crystal (−)-bisnoreseroline structure (light green, in big-ball-and-stick) on the pharmacophoric (−)-bisnorphysostigmine (**3**, magenta, in ball-and-stick) conformation; (**c)** The pharmacophore model fitted into the active site of AChE (PDB code 3ZV7). (Pharmacophoric features: green, HBA; orange, RA; red, PI; cyan, HYD; gray, Xvol; Active site residues were shown as gray lines; Key residues mapped to the excluded volumes were highlighted as yellow sticks.)

To extend our knowledge of the pharmacophore model from ligand basis to its interactive target, all model features were fitted into AChE active site. A recent study on the X-ray crystal structure of a complex of (−)-bisnorcymserine (Figure 3(a)) and AChE (PDB code 3ZV7)^45^ had revealed that a leaving group, (−)-bisnoreseroline (Figure 3(a)), was trapped in the catalytic site. The binding pose of (−)-bisnorphysostigmine (**3**) was supposed similar to that of (−)-bisnorcymserine since they had a common leaving group. Atom coordinates of the crystal AChE structure were fitted to those of the pharmacophore model through heavy atom superimposition of crystal (−)-bisnoreseroline structure upon pharmacophoric (−)-bisnorphysostigmine (**3**) conformation (Figure 3(b)). Figure 3(c) showed mapping of the pharmacophore model into the active site of AChE. Residues involved in inhibitor-enzyme interactions were represented as gray lines. Interestingly, two excluded volumes in the model were aligned with two catalytic triad residues, Ser200 and His440, highlighted as yellow sticks in Figure 3(b,c). HBA was located nearby the hydroxyl of Ser200, wherein covalent bonds were to be formed via carbamoylation. RA feature formed hydrophobic interactions with the backbone of Gly118 and the side chain of His440. PI feature was surrounded by Phe330 and Trp84, also known as the anionic site. The HYD feature near PI showed week interactions with Gly117 and Tyr130. These results verified the reliability of the pharmacophore model in depth, and strengthened its predictive power for carbamate-type pseudo-irreversible AChE inhibitors.

### Pharmacophore-based design and synthesis

As we early reported, (−)-meptazinol is a moderate AChE inhibitor (IC_50_ 41 *µ*M^17^, Table 4) and it binds the enzyme by reversible mechanism. Guided by the pharmacophore model generated above, we selected (−)-meptazinol as the scaffold to build carbamoyl groups on. (−)-Meptazinol dimethylcarbamate (**42**) and phenylcarbamate (**43**) (Figure 1) were designed by carbamoylating the phenolic hydroxyl of (−)-meptazinol. They were supposed to be good AChE inhibitors for their perfect superimposition upon the model.

**Figure 4.**
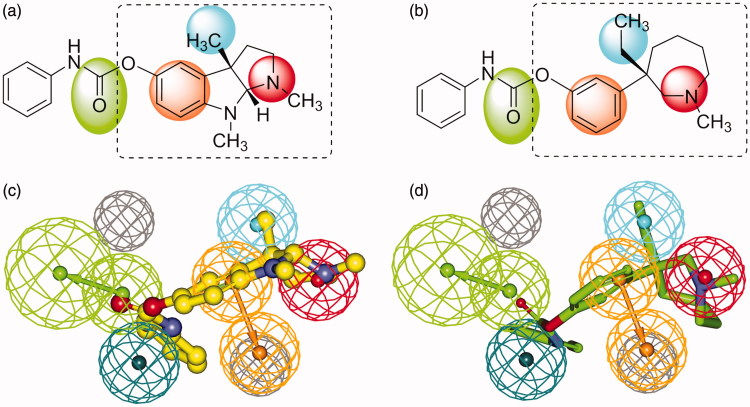
2D structures of phenserine (**a**) and (−)-meptazinol phenylcarbamate (**b**) with color backgrounds highlighting common chemical features, and 3D overlays of phenserine (**c**, yellow, in ball-and-stick) and (−)-meptazinol phenylcarbamate (**d**, green, in stick) with the best pharmacophore model. (Pharmacophoric features: green, HBA; orange, RA; red, PI; cyan, HYD; gray, Xvol.)

Figure 4 showed the geometrical structural similarity between phenserine (Figure 4(a)) and (−)-meptazinol phenylcarbamate (**43**) (Figure 4(b)), and the spatial overlapping of phenserine (Figure 4(c)) and **43** (Figure 4(d)) with the pharmacophore model. Although built on azapane scaffold, **43** matched four of the five features in the model just as phenserine did, especially at the very important PI (red) center and HYD (cyan) region. The only nitrogen in azapane ring of **43** resembled the N^1^-nitrogen of phenserine. Mimicking the (3aS)-methyl group presented in phenserine, (3S)-ethyl group of **43** occupied the HYD feature.

AChE inhibitory activities of **42** and **43** were predicted according to ligand pharmacophore mapping, giving an estimated IC_50_ of 100 nM for **42** and 560 nM for **43** by BEST algorithm (Table 3). To our knowledge, conformational analysis of (−)-meptazinol derivatives is complicated due to the seven membered ring system. Our early NMR studies^46^ on (−)-meptazinol hydrochloride had revealed that two stable conformers were detected in solution, including a lowest energy conformer with phenol group in equatorial orientation and a less favorable energy conformer with phenol group in axial orientation. A conformer in axial orientation was also found in the X-ray crystal structure of bis(9)-(−)-*nor*-meptazinol/AChE complex (PDB code 2W6C)^18^. It was therefore believed that pharmacophoric conformer of (−)-meptazinol might be the conformer in axial orientation with less favorable energy. To avoid energy minimization in the conformation generation step and to explore more ring conformations, CAESAR algorithm was performed to **42**, **43** and (−)-meptazinol. As a result, (−)-meptazinol was predicted an IC_50_ of 18 *µ*M, similar to the experimental activity. By CAESAR algorithm, the estimated IC_50_s of **42** and **43** were 75 nM and 370 nM, respectively (Table 3), and the conformation of **43** fitted to the pharmacophore model was in axial orientation (Figure 4(d)).

**Table 3.** Predicted AChE inhibitory activities of **42**, **43** and (−)-meptazinol by BEST and CAESAR algorithms.

	BEST algorithm		CAESAR algorithm	
Compounds	Estimated IC_50_ (nM)	Fit value	Estimated IC_50_ (nM)	Fit value
**42**	100	8.10	75	8.23
**43**	560	7.36	370	7.54
(−)-Meptazinol	3300	6.59	18 000	5.86

**Table 4.** AChE and BChE inhibitory activities, selectivity and acute toxicity of (−)-meptazinol carbamates, compared with classical carbamate-type AChE inhibitors.

	IC_50_ ± SEM (nM)			
Compounds	AChE^a^	BChE^b^	Selectivity for AChE^c^	LD_50_ (mg/kg)
**42**	6.93 ± 2.45	3.17 ± 1.34	0.46	12 (p.o.); 1.4 (i.p.)
**43**	31.6 ± 3.5	67.1 ± 23.7	2.1	73 (p.o.); 45 (i.p.)
(−)-Meptazinol	41 000 ± 14 000^d^	15 000 ± 4000^d^	0.37	N/A^e^
Rivastigmine	5460 ± 1470	1590 ± 38	0.29	3-6 (p.o.)^f^
Physostigmine	27.9 ± 2.4^g^	16.0 ± 2.9^g^	0.57	4.5 (p.o.)^h^
Phenserine	24.0 ± 6.0^g^	1300 ± 85^g^	54	25 (i.p.)^i^

a Mice brain homogenate was the source of AChE unless otherwise indicated.

b Mice serum was the source of BChE unless otherwise indicated.

c Selectivity for AChE: IC_50_ for BChE divided by IC_50_ for AChE.

d See reference^17^.

e N/A: not available.

f See reference^53^.

g Human erythrocyte AChE and human serum BChE were used, see reference^22^.

h See reference^54^.

i See reference^55^. p.o.: oral administration; i.p.: intraperitoneal administration.

**Scheme 2.**
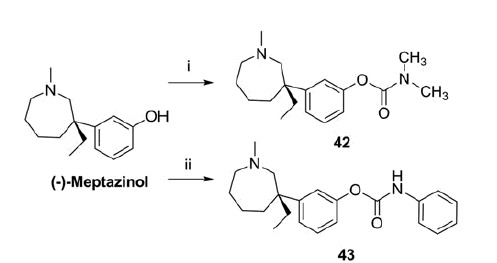
Synthesis of **42** and **43**.^a^ ^a^Reagents and conditions: (i) Me_2_NCOCl, NaH, dry THF, 0 °C to rt, 2 h, 100%; (ii) PhNCO, Na, dry Et_2_O, rt, 3 h, 72%

Methodology employed for the synthesis of **42** and **43** was illustrated in Scheme 2. **42** was synthesized in quantitative yield by treating (−)-meptazinol with N,N-dimethylcarbamoyl chloride in the presence of sodium hydride at room temperature. The coupling of (−)-meptazinol with phenyl isocyanate at room temperature in the presence of sodium gave **43** in 72% yield. **42** and **43** were prepared as hydrochloride salts for the following *in vitro* and *in vivo* assays. Structures of the hydrochloride salts were characterized by [*α*]_D_, IR, ^1^H NMR, ^13^C NMR, MS, and elemental analysis.

### Cholinesterase inhibitory potency and selectivity

The synthesized carbamate compounds **42** and **43** were tested *in vitro* for AChE/BChE inhibitory potency and selectivity (Table 4). Mice brain homogenate and mice serum were used as sources of AChE and BChE, respectively. The dimethylcarbamate **42** inhibited AChE with IC_50_ value of 6.93 nM, about 790 times lower than that of rivastigmine (IC_50_ 5460 nM). The phenylcarbamate **43**, showing an IC_50_ of 31.6 nM, was about 170 times more potent than rivastigmine and almost five times less potent than **42**. Compared with the parent compound (−)-meptazinol, **42** and **43** showed a 5900-fold and 1300-fold increase, respectively, in the inhibition of mice brain AChE. With regard to activities reported by Yu et al.^22^, **42** was four times more potent than physostigmine (IC_50_ 27.9 nM), while **43** was 1.3 times less potent than phenserine (IC_50_ 24.0 nM).

As for selectivity, **42** was slightly more selective (twofold) to BChE similar to rivastigmine and physostigmine, while **43** was an AChE-selective inhibitor, showing a twofold selectivity for AChE versus BChE (IC_50_ 67.1 nM). Similar to phenserine, **43** would have less peripheral side effects and lower acute toxicity than those BChE-selective carbamates, such as physostigmine and **42**.

### Mechanism of enzyme inhibition and kinetic parameters

Understanding of potent AChE inhibitors’ mechanism of action and kinetic parameters is key information to establish the structure-activity relationship and design new compounds for the treatment of AD. The characteristics of AChE activity inhibition by **42** and **43** were revealed by enzyme kinetics assays. The plots of residual enzyme activity versus enzyme concentration at different concentrations of **42** and **43** gave a family of straight lines with a *y*-axis intercept, suggesting that both compounds were reversible AChE inhibitors. Their enzyme inhibitory properties were further modeled using double-reciprocal plots. Variance of the velocity of control group could be explained as the degradation of enzyme. Increasing the concentrations of **42** and **43** led to a decrease in *V*_max_ and an unvaried *K*_m_ (*x*-intercepts) (Table 5). **42** decreased the *V*_max_ by 18% and 29% at the concentrations of 100 and 250 nM and **43** decreased the *V*_max_ by 10% and 25% at the concentrations of 25 and 50 nM, consistent with the typical characteristics of uncompetitive inhibitors. The observed results showed that both inhibitors bound only to enzyme–substrate complex, not the free enzyme.

**Table 5.** *K*_m_ and *V*_max_ values of **42** and **43** on rHuAChE.

Compounds	Concentration (nM)	*K*_m-app_ (μM)	*V*_max-app_ (μM/min)
**42**	0	112.67	34.94
	100	113.97	28.73
	250	115.10	24.70
**43**	0	147.11	19.92
	25	145.87	17.84
	50	143.90	14.94

The inhibition of AChE by carbamates involves carbamoylation of the enzyme and production of a covalent adduct. The carbamoylated enzyme is then hydrolyzed to regenerate the free enzyme. The process is time-dependent, therefore, determination of the kinetic parameters is of utmost importance to assess time of action. Bartolini et al.^47^ has reported that AChE immobilized disk, which could maintain enzymatic activity for about 2 months, was a powerful tool to evaluate both the carbamoylation and the decarbamoylation constants in single experiment. In this study, the well-known pseudo-irreversible AChE inhibitor physostigmine (**1**) was first selected as a reference compound to verify the reliability of our disk. Percent inhibition of enzyme activity [(*A*_0_−*A*_i_)/*A*_0_×100%] was plotted versus time. The curve was fitted to Perola’s mathematical equation. The calculated *k*_i_, *k*_3_, and *K*_D_ of physostigmine were (4.78 ± 1.13) × 10^5^ M^−1 ^min^−1^, (1.94 ± 0.36) × 10^−2 ^min^−1^ and (4.09 ± 0.22) × 10^−8^ M^–1^, respectively, consistent with the previously reported results^43^^,^^48–50^, indicating the AChE-immobilized disk we prepared was robust for the determination of kinetic constants of **42** and **43**.

**Figure 5.**
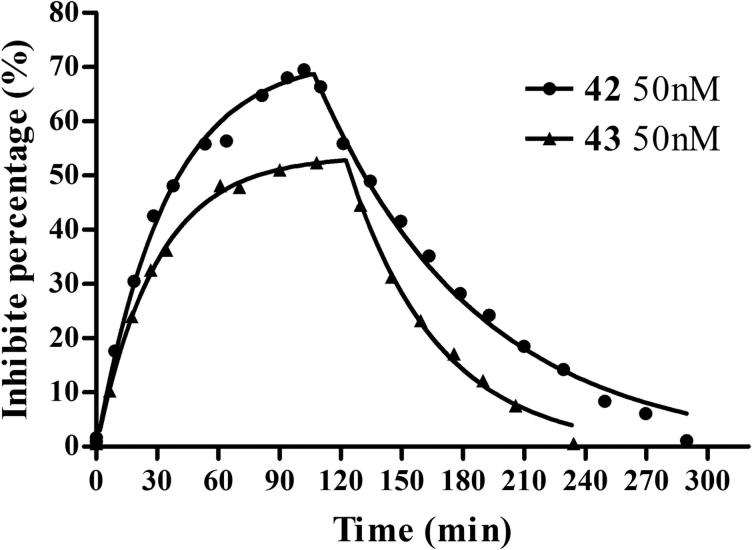
AChE-CIM-IMER time-dependent carbamoylation (inhibitors in buffer A) and decarbamoylation (buffer A as mobile phase) of AChE by **42** (50 nM) and **43** (50 nM). The percent of inhibition measured is equal to the percent of carbamylated enzyme. The curve was fitted to Perola’s mathematical equation^43^.

The data of the carbamoylation and decarbamoylation of AChE by **42** and **43** fitted well to Perola’s equation^42^. Figure 5 showed that the immobilized AChE in EDA CIM disk was time-dependently inactivated by **42** and **43** at 50 nM. The carbamoylation half-times of **42** and **43** were found to be 23.5 min and 20.3 min, respectively, longer than that of physostigmine (3.9 min) and rivastigmine (11.4 min) reported by Bartolini et al.^39^. About 2 h and 3 h flushing were required to achieve a complete recovery of AChE activity after complete inhibition by **42** and **43,** respectively, similar to physostigmine (2 h) but much shorter than rivastigmine (34 h). The *k*_i_, *k*_3_ and *K*_D_ values of **42** and **43** are shown in Table 6. **43** could bind to and dissociated from AChE faster than **42**, indicating that the enzyme was more affinitive to **42** than **43**. And, **42** inhibited AChE more strongly than **43**. There was no obvious difference of the *k*_i_ and *k*_3_ values between **42** and **43**. The enzyme affinity, carbamoylation and decarbamoylation rates and the duration of the inhibition of **42** and **43** were similar to that of physostigmine, suggesting that both the compounds reacted with the enzyme as a pseudo-irreversible inhibitor, in a way typical to carbamates, through quick formation of an addition complex and subsequent slow decarbamoylation.

**Table 6.** Kinetic constants of **42** and **43** on rHuAChE (Mean ± SD, *n* = 6).

	*K*_D_ (M^−1^)	*k*_i_ (M^−1^min^−1^)	*k*_3_ (min^−1^)
**42**	(3.02 ± 0.51) × 10^−8^	(4.47 ± 1.21) × 10^5^	(1.35 ± 0.31) × 10^−2^
**43**	(1.08 ± 0.30) × 10^−7^	(2.12 ± 0.46) × 10^5^	(2.18 ± 0.12) × 10^−2^

### Anti-amyloidogenic properties in SH-SY5Y-APP_695_ cells

**Figure 6.**
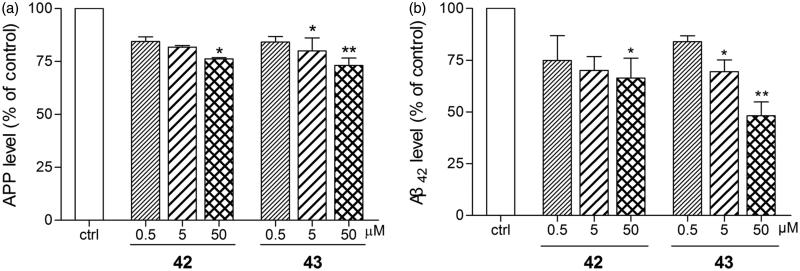
Anti**-**amyloidogenic properties of **42** and **43** were evaluated in SH-SY5Y-APP_695_ cells. **42** and **43** (0.5, 5 or 50 *µ*M) were applied to SH-SY5Y-APP_695_ for 16 h incubated at 37 °C in 5% CO_2_. The cells and the culture medium were prepared to immunofluorescence and ELISA assay, respectively. (**a**) **42** and **43** mediated APP levels examined by immunofluorescence assay on KineticScan HCS System. (**b**) **42** and **43** mediated Aβ_42_ levels examined by ELISA assay with Human/Rat *β-*Amyloid (42) ELISA Kit Wako. The values are the mean ± SEM of three independent measurements. *p < 0.05, **p < 0.01 significant difference from untreated SH-SY5Y-APP_695_.

Anti-amyloidogenic properties of **42** and **43** were evaluated with HCS in SH-SY5Y-APP_695_ cells^43^. After exposure to 50 µM of **42** and **43** for 16 h, intracellular APP levels were markedly reduced by 23.5% and 26.9%, respectively (Figure 6(a)). Phenserine was reported to produce a 40% decrease of APP level at the same concentration^51^. At a lower concentration of 5 *µ*M, **43** exhibited a 20.0% decrease of APP levels (Figure 6(a)).

Although APP over-expression is a risk factor to AD, APP is still necessary to maintain normal physiological function. So, it will be more important to evaluate anti-amyloidogenic properties by determining Aβ lowering effects, especially the neurotoxic form Aβ_42_. Application of 50 µM of **42** and **43** to SH-SY5Y-APP_695_ cells remarkably reduced the level of Aβ_42_ by 34.0% and 51.9%, respectively (Figure 6(b)), keeping the level of less toxic Aβ_40_ unchanged (data not shown). The Aβ lowering effect of **43** was better than that of phenserine (a 31% decrease of total Aβ^14^) at the concentration of 50 µM. Even at a lower concentration of 5 µM, **43** still produced a 30.5% decrease of the Aβ_42_ level (Figure 6(b)).

The actions of **42** and **43** on reducing APP and Aβ_42_ levels were very promising, but the mechanism was still complicated. Most AChE inhibitors produce Aβ lowering effects by altering APP into non-amyloidogenic pathway^52^. This anti-amyloidogenic property results from post-receptor effects, such as Protein Kinase Cα (PKCα) activation, of the increased ACh level secondary to AChE inhibition. Exhibiting poorer AChE inhibition, **43** produced better APP and Aβ_42_ lowering properties in comparison with **42**, which indicated additional non-cholinergic involvement in the anti-amyloidogenic effect of **43**. Phenserine was reported to reduce the levels of APP and Aβ via a non-cholinergic mechanism by downregulating the translation of APP mRNA14. Although **43** was less potent than phenserine in reducing APP level, its ability to reduce Aβ, especially the most neurotoxic Aβ_42_, was much higher than phenserine. It was possible yet still a hypothesis that **43** might have a direct action on the amyloidogenic processing pathway. Further experiments were still needed to clarify the mechanism.

### Acute toxicity

The LD_50_ values of **42** and **43** were tested in mice after intraperitoneal (i.p.) and oral (p.o.) administration, and corresponding results are reported in Table 4. As the doses of **42** and **43** escalating, peripheral cholinergic side effects such as salivation, twitch, and incontinence were observed. **42** showed high acute toxicity (LD_50_ 1.4 mg/kg) after i.p. administration. When administered orally, **42** (LD_50_ 12 mg/kg) was almost three times less toxic than physostigmine (LD_50_ 4.5 mg/kg)^54^, although **42** showed four times higher potency than physostigmine in *in vitro* test. The LD_50_ of **43** (73 mg/kg, p.o.) was 12–24 times higher than that of rivastigmine (3–6 mg/kg, p.o.)^53^. If administered intraperitoneally, **43** (LD_50_ 45 mg/kg) was slightly less toxic compared with phenserine (25 mg/kg)^55^. Thus, **43** showed low acute toxicity and deserved further studies in cholinergic impairment animal models to evaluate its *in vivo* cognitive enhancement function.

## Conclusions

In summary, (−)-meptazinol carbamate derivatives were designed based on a 3D pharmacophore model built using 3D QSAR Pharmacophore Generation module in DS from 25 known carbamate-type AChE inhibitors. The best pharmacophore model consists of five chemical features (namely HBA, RA, PI, and two HYDs) and two excluded volumes. The existence of a HYD region near the PI feature have been recognized as essential chemical characteristics in the model to differentiate enantiomers. Merging of carbamoyl groups onto the (−)-meptazinol scaffold generated new bifunctional ligands with dual actions on both cholinesterase and amyloidogenic pathways.

The synthesized compounds **42** and **43** were verified as nanomolar cholinesterase inhibitors in *in vitro* assay. **42** and **43** showed uncompetitive inhibition and reacted with the enzyme as a pseudo-irreversible inhibitor, such as typical carbamates, through quick carbamoylation and subsequent slow decarbamoylation. **42** (IC_50_ 6.93 nM) was more potent in inhibiting AChE than **43**, and was slightly selective to BChE (two-fold). In acute toxicity test, **42** had lower LD_50_ values (12 mg/kg, p.o.) and showed more peripheral cholinergic side effects. However, the phenylcarbamate **43** was more promising and exhibited significant anti-cholinesterase and anti-amyloidogenic properties.

**43** (IC_50_ 31.6 nM) was 170 times more potent than rivastigmine in inhibiting AChE, and was 1.3 times less potent than phenserine. **43** exhibited a twofold selectivity for AChE, therefore milder peripheral side effects and lower acute toxicity were observed for **43** (LD_50_ 73 mg/kg, p.o.). **43** also showed Aβ lowering effects (51.9% decrease of Aβ_42_) superior to phenserine (31% decrease of total Aβ) at the concentration of 50 µM. Even at a lower concentration of 5 µM, **43** still reduced APP level by 20.0% and reduced Aβ_42_ by 30.5%. The dual actions of cholinesterase inhibition and anti-amyloidogenesis indicated a potential use of **43** as symptomatic and disease-modifying agent for the treatment of AD, which deserved further studies in cholinergic impairment animal models.
